# Marrow Adiposity Content and Composition Are Not Associated With Incident Fragility Fractures in Postmenopausal Women: The ADIMOS Fracture Study

**DOI:** 10.1210/jendso/bvaf033

**Published:** 2025-02-24

**Authors:** Cécile Philippoteaux, Sammy Badr, Daniela Lombardo, Emeline Cailliau, Stefan Ruschke, Dimitrios C Karampinos, Anne Cotten, Julien Paccou

**Affiliations:** Department of Rheumatology, Univ. Lille, CHU Lille, F-59000 Lille, France; Department of Radiology and Musculoskeletal Imaging, Univ. Lille, CHU Lille, MABlab ULR 4490, F-59000 Lille, France; Department of Rheumatology, Univ. Lille, CHU Lille, F-59000 Lille, France; Biostatistics Department, CHU Lille, F-59000 Lille, France; Department of Diagnostic and Interventional Radiology, Klinikum rechts der Isar, School of Medicine, Technical University of Munich, 81675 Munich, Germany; Department of Diagnostic and Interventional Radiology, Klinikum rechts der Isar, School of Medicine, Technical University of Munich, 81675 Munich, Germany; Department of Radiology and Musculoskeletal Imaging, Univ. Lille, CHU Lille, MABlab ULR 4490, F-59000 Lille, France; Department of Rheumatology, Univ. Lille, CHU Lille, MABlab ULR 4490, F-59000 Lille, France

**Keywords:** osteoporosis, fracture, bone marrow adipose tissue, bone mineral density

## Abstract

**Context:**

Noninvasive measurement of bone marrow adipose tissue using magnetic resonance imaging and proton density fat fraction (PDFF) may enhance clinical fractures prediction in postmenopausal women.

**Objective:**

This study aimed to assess the association between PDFF measurements and clinical fracture incidence.

**Methods:**

A longitudinal study was conducted. Postmenopausal women with recent osteoporotic fractures (<12 months) and with osteoarthritis without fractures were included. Lumbar spine and proximal femur PDFFs were measured at baseline using water-fat imaging (WFI) and dual-energy x-ray absorptiometry scans. Clinical fractures were recorded during follow-up.

**Results:**

Among 195 participants (mean age 67.4 ± 10.0 years, body mass index 27.2 ± 5.9 kg/m²), the PDFF (WFI-based) was higher at the proximal femur, particularly at the femoral head (90.0% ± 4.9%), compared to the lumbar spine (57.8% ± 9.6%). Over a mean follow-up period of 37.2 ± 11.6 months, 7 participants died, 29 (14.9%) experienced incident clinical fractures, and 1 was lost to follow-up. The lack of an association between WFI-based PDFFs and the incidence of clinical fractures was demonstrated regardless of the region of measurement (hazard ratio [HR] = 0.95 [95% CI 0.67-1.35], *P* = 0.77 at the lumbar spine, HR = 1.07 [95% CI 0.71-1.63], *P* = 0.74 at the femoral neck). Stepwise regression analysis did not alter these findings, and the variable “recent osteoporotic fractures” was found to be significantly associated with incident clinical fractures.

**Conclusion:**

This study found no evidence of a relationship between PDFF and clinical fracture incidence in postmenopausal women. Further studies are necessary involving larger cohorts and longer follow-up periods.

Bone marrow adipose tissue (BMAT) is found within the bone cavity and is a component of the bone marrow (BM) [1]. BMAT is derived from bone marrow stromal cells (BMSCs), which can differentiate into various cell types including bone marrow adipocytes (BMAds) [2, 3]. The distribution of BMAT throughout the skeleton changes with age, starting with red BM, which is mostly composed of hematopoietic cells at birth [4]. In adulthood, these cells are replaced by yellow BM containing BMAds in the distal regions of the appendicular skeleton, accounting for approximately 70% of the total BM volume [5]. BMAT is distinct because it is the only tissue where adipocytes and bone cells are located side by side [6].

The relationship between BMAT and bone health has become an exciting area of research, owing to the development of BMAT imaging [7, 8]. Magnetic resonance imaging (MRI) is considered a go-to, noninvasive imaging method for measuring in vivo BMAT in humans [9, 10]. Although proton magnetic resonance spectroscopy (¹H-MRS) is considered to be the gold standard, water-fat imaging (WFI) is now viewed as an efficient more convenient alternative [9, 10]. The International Bone Marrow Adiposity Society (BMAS) has recently released recommendations to standardize imaging protocols, with the goal of increasing comparability across studies and different sites' measures [11]. Several cross-sectional studies have used MRI in conjunction with dual-energy x-ray absorptiometry (DXA) to show that higher BMAT levels are associated with lower bone mineral density (BMD) and a higher prevalence of vertebral fractures (VF) [12-14].

Limited prospective data are currently available on the lipid content, composition, and its relationship with bone health, including BMD and fracture outcomes [15, 16]. In the AGES-Reykjavik study, no associations were found between bone marrow fat fraction (BMFF) measured at the lumbar spine, incident radiographic VFs, and incident clinical fractures in either men or women [15]. However, the authors discovered a connection between higher levels of unsaturated marrow lipids and a reduced risk of incident radiographic VFs and a reduced risk of incident clinical fractures in men, but not in women [16]. Further longitudinal studies are necessary to better understand the relationship between BMAT, BMD, and fracture outcomes. In addition, measurements of lipid content and composition at the proximal femur are required in accordance with the guidance from the BMAS [11].

In a longitudinal study, we used data from the ADIMOS cohort [17] to test the hypothesis that noninvasive measurements of lipid content and composition using MRI are associated with incident clinical fractures. Our objective was to assess the association between lipid content, composition, and fracture outcomes in postmenopausal women aged 45 to 95 years.

## Methods

### Study Protocol

The ADIMOS study is a case-control investigation (clinicaltrials.gov ID no. NCT03219125) designed to explore the relationship between imaging-based proton density fat fraction (PDFF) and recent osteoporotic fractures in postmenopausal women at the lumbar spine and proximal femur [17]. The study selected postmenopausal women with recent osteoporotic fractures (less than 12 months old) as cases (N = 100), and postmenopausal women with osteoarthritis and no history of fragility fractures as controls (N = 99). The exclusion criteria were chronic kidney disease with a calculated creatinine clearance of less than 30 mL/min/1.73 cm², contraindications to MRI, and current use of substances known to affect BMAT, such as oral glucocorticoids, anti-osteoporosis medications (bisphosphonates, denosumab, raloxifene, or teriparatide), thiazolidinediones, and hormone replacement therapy. However, prior use of anti-osteoporosis medications (AOM) and hormone replacement therapy over a period of 12 months was allowed.

This ancillary study was approved by the local Institutional Review Board and all participants provided written informed consent before participation.

### Study Participants

Postmenopausal women aged 45 to 95 years were recruited from the Lille University Hospital, Lille, France, between October 2018 and June 2021. The ADIMOS study documented the risk factors for osteoporosis, including current smoking status, excessive alcohol consumption, and prior oral corticosteroids use. Furthermore, information was collected on the Charlson Comorbidity Index (CCI), leisure time activities scored on a scale of 0 to 15, and medication use.

### Study Procedure

To perform this ancillary study, all participants were contacted by phone between January 2023 and October 2023 to complete an evaluation questionnaire performed by a rheumatologist. When patients were unable to answer the questions, a family member or general practitioner was interviewed by the same rheumatologist. The questionnaire included closed-ended questions: (i) new incident fracture; (ii) death; and (iii) actual initiation of AOM or failure to initiate the prescribed treatment. If a new fracture was reported, the rheumatologist gathered additional details, including the mechanism, trauma energy, fracture location, and asked if any imaging was performed for assessment.

### Imaging-Based Proton Density Fat Fraction

MRI scans were conducted on a 3-T full-body scanner (Ingenia; Philips Medical Systems, Best, The Netherlands) using water-fat imaging (WFI) sequences (mDixon-Quant; Philips Healthcare, Best, The Netherlands) for BMAT quantification. The PDFF maps were calculated offline using a precalibrated 7-peak fat spectrum and a single T2* correction. For each subject, the average PDFF value was measured at the lumbar spine (L1–L4, excluding severe structural changes and fractured vertebrae) and the nondominant hip (femoral head, neck, and proximal diaphysis).

Assessment of BMAT composition was performed using a single ¹H-MRS voxel placed in the L3 vertebral body (or L2 if the L3 vertebral body was fractured) and the femoral neck of the nondominant hip, avoiding the cortical bone. Post-processing was carried out using ALFONSO (A versatile Formulation fOr N-dimensional Signal model fitting of MR spectroscopy data) scripts written in MATLAB, version R2022a (MathWorks) [18]. The fitting strategy involved using common T2 values and linewidth constraints across all 10 fat peaks. PDFF was calculated as the percentage of fat signal relative to the total signal intensity (fat + water). The apparent lipid unsaturation level (aLUL) was determined using the olefinic peak (UL) as the most representative unsaturated lipid: aLUL (%) = UL/total fat.

The detailed protocol of MRI acquisitions was previously described in the princeps article [17].

### Laboratory Measurements

Blood samples were collected from all patients after overnight fasting. Standard tests were performed to determine the total calcium, phosphorus, and high-sensitivity C-reactive protein (hs-CRP) levels. The levels of intact parathyroid hormone (PTHi) were measured using chemiluminescent immunoassay on an automatic analyzer (Architect, Abbott Laboratories, USA). Levels of 25-OH vitamin D were determined using a competitive chemiluminescent immunoassay on an IDS-iSYS device (IDS, Pouilly en Auxois, France). The levels of procollagen I intact N-terminal (PINP) and serum cross-laps (CTX) were measured using a chemiluminescence assay on an IDS-iSYS Multi-Discipline Automated Analyzer (Immunodiagnostic Systems, Inc., Fountain Hills, AZ, USA).

### BMD Measurements

Bone mineral density was evaluated at the lumbar spine (L1–L4) and nondominant hip using DXA (HOLOGIC Discovery A S/N 81360). The data collected included BMD measurements (in grams per square centimeter of hydroxyapatite) at 3 specific sites: lumbar spine, total hip, and femoral neck. In accordance with the World Health Organization recommendations, osteoporosis was diagnosed if the T-score ≤ −2.5.

### Statistical Analyses

Categorical variables are expressed as numbers (percentages). Continuous variables are expressed as mean (SD) in the case of normal distribution or median [interquartile range]. Normality of distribution was assessed using histograms and the Shapiro-Wilk test. The cumulative incidence of clinical fractures was estimated using the Kalbfleisch and Prentice method, considering death as a competing event. The association of PDFFs using WFI-based MRI with cumulative clinical fracture incidence was evaluated using the Fine and Gray regression model. Hazard ratios (HR) on the subdistribution of clinical fractures and their 95% CI were derived from the models as effect sizes. In order to consider predefined confounding factors (age, BMD [lumbar spine or total hip]), CCI, number of falls in the last 12 months, and recent osteoporotic fracture [less than 12 months old]) and to account for the small number of events, the multivariable Fine and Gray regression model was performed using a forward stepwise approach to select confounders, using a selection criterion of *P* < .20. The association of ¹H-MRS PDFF and ¹H-MRS aLUL values with cumulative clinical fracture incidence was evaluated using the same method described above. Proportional hazard assumptions for variables included in the Fine and Gray regression models were assessed by examining the Schoenfeld residual plots, while for quantitative variables, the log-linearity assumption was assessed by examining the Martingale residual plots.

Statistical testing was conducted using a 2-tailed α level of 0.05. Data were analyzed using the SAS software (version 9.4; SAS Institute, Cary, NC, USA).

## Results

### Baseline Characteristics

Follow-up information was available for 195 of 199 participants. Table 1 summarizes the baseline characteristics of the participants. The mean age of the 195 participants was 67.4 ± 10.0 years and the mean body mass index (BMI) was 27.2 ± 5.9 kg/m². The mean CCI was 3.0 ± 2.3, and the most frequent comorbidities were nonmetastatic cancer (n = 38; 19.5%) and type 2 diabetes mellitus (n = 24; 12.4%). Regarding clinical risk factors for osteoporosis, current smoking was found in 23 participants (11.8%) and excessive alcohol consumption was recorded for 11 patients (5.6%). The mean 25(OH)vitamin D level was 28.4 ± 11.5 ng/mL. Regarding the fat content (imaging-based) data, PDFF was higher at the proximal femur than in the lumbar spine (57.8% ± 9.6%), particularly at the femoral head (90.0% ± 4.9%).

**Table 1.** Participants' general characteristics and biochemistry results at baseline

	N	Participants (n = 195)
Age [years]	195	67.4 ± 10.0
Weight [kg]	195	69.9 ± 15.8
Height [cm]	195	160.2 ± 6.6
BMI [kg/m^2^]	195	27.2 ± 5.9
Leisure time activity (score 0-15)	192*^b^*	9.0 ± 2.5
Population details (fracture/osteoarthritis)*^a^*	195	97*^a^* (49.7)/98*^a^* (50.3)
Comorbidities		
Nonmetastatic cancer	195	38 (19.5)
Type 2 diabetes	195	24 (12.4)
Chronic pulmonary disease	195	14 (7.2)
Stroke or TIA	195	10 (5.1)
Charlson Comorbidity Index	195	3 (2 to 4)
Clinical risk factors of osteoporosis		
Excessive alcohol consumption	195	11 (5.6)
Current smoking	195	23 (11.8)
Family history of hip fracture	195	22 (11.3)
Previous use of corticosteroids	195	9 (4.6)
Number of falls in the last 12 months	195	0 (0 to 1)
Biochemistry results		
Hs-CRP [mg/L]	195	3.0 (3.0 to 5.0)
Calcium [mmol/L]	195	2.4 ± 0.1
25(OH) vitamin D [ng/mL]	195	28.4 ± 11.5
Serum PTH [pg/mL]	195	44.0 (34.0 to 57.0)
Creatinine [µmol/L]	195	62.0 (53.0 to 71.0)
Creatinine clearance (MDRD formula) [mL/mn]	195	85.0 (72.2 to 95.0)
PINP [ng/mL]	188^*c*^	62.0 (45.0 to 79.0)
CTX [pmol/L]	194^*d*^	3330 (2169 to 4826)
Bone mineral density		
BMD lumbar spine (g/cm^2^)	194^*e*^	0.847 ± 0.169
BMD total hip (g/cm^2^)	192^*f*^	0.757 ± 0.135
BMD femoral neck (g/cm^2^)	192^*f*^	0.632 ± 0.127
Fat content (imaging-based) DIXON		
PDFF lumbar spine (%)	195	57.8 ± 9.6
PDFF femoral head (%)	188^*g*^	90.0 ± 4.9
PDFF femoral neck (%)	188^*g*^	81.8 ± 8.3
PDFF femoral diaphysis (%)	188^*g*^	80.5 ± 9.2
Fat content (imaging-based) ^1^H-MRS		
L3 PDFF (%)	113*^h^*	57.4 ± 11.2
L3 aLUL (%)	113*^h^*	4.3 ± 0.6
Femoral neck PDFF (%)	115*^i^*	80.8 ± 9.5
Femoral neck aLUL (%)	115*^i^*	3.7 ± 0.7

Values expressed as numbers (%), mean ± SD or median (IQR).

Abbreviations: aLUL, apparent lipid unsaturation level; BMD, bone mineral density; BMI, body mass index; CTX, collagen type 1 cross-linked C-telopeptide; ^1^H-MRS, proton magnetic resonance spectroscopy; hs-CRP, high-sensitivity C-reactive protein; IQR, interquartile range; PINP, procollagen type 1 N-terminal propeptide; PDFF, proton density fat fraction; PTH, parathyroid hormone; TIA, transient ischemic attack.

^*a*^The population originates from the ADIMOS case-control study, comprising 100 postmenopausal women with recent fractures (cases) and 99 with osteoarthritis and no fractures (controls). Follow-up data were available for 195 participants: 97 from the cases and 98 from the controls.

^*b*^Data on leisure time activity was not collected in 3 patients.

^*c*^Serum PINP measurements were not performed in 7 patients.

^*d*^Serum CTX measurement was not performed in 1 patient.

^*e*^Lumbar spine BMD measurements were not performed in 1 woman (vertebral fractures at L1, L2, and L3).

^*f*^Hip BMD measurements were not available in 3 women (bilateral hip arthroplasty).

^*g*^Hip PDFF measurements were not available in 7 women (bilateral hip osteonecrosis, n = 1; bilateral hip arthroplasty, n = 3; unacceptable quality of measurements, n = 3).

^*h*1^H-MRS was performed at the L3 vertebral level in a subgroup of 113 participants in the princeps study.

^*i*1^H-MRS was performed at the femoral neck in a subgroup of 115 participants in the princeps study.

### Anti-Osteoporosis Medication

The follow-up questionnaire revealed that AOM was prescribed to 78 out of 195 participants (40.0%), in alignment with French clinical guidelines [19] We remind that none of the participants received an AOM at baseline of the ADIMOS study. The most commonly prescribed AOM was zoledronic acid, administered to 52 patients (66.7%). Teriparatide was prescribed to 18 patients (23.1%), oral bisphosphonates to 5 patients (6.4%), and denosumab to 3 patients (3.8%).

### Follow-Up

Among the 195 participants analyzed (mean follow-up of 3.1 years), 7 died and 1 was lost to follow-up. A total of 29 patients (10 in the control group and 19 in the case group) experienced incident clinical fractures, with a cumulative incidence of 18.4% (95% CI 11.7 to 26.3); some participants presented multiple fractures (Table 2). Among the participants, 8 were identified with at least one clinical VF, 13 with upper limb fractures, 4 with lower limb fractures, and 4 with another type of fracture (including rib fractures). Additionally, 158 participants did not present any event at the end of the follow-up period.

**Table 2.** Incident clinical fractures during the follow-up period

N	Age at baseline (years)	Fracture at baseline	Follow-up (months)	Type of incident clinical fractures
**1**	52	0	19	Hand (scaphoid fracture)
**2**	74	1 (pelvis fracture)	48	Shoulder (scapula fracture)
**3**	65	1 (L2)	18	Wrist (radius fracture)
**4**	68	1 (T7, T11, and L5)	48	Hip fracture
**5**	79	1 (T6, T7, T8, T11, and L4)	31	Vertebrae (T12)
**6**	58	1 (T7, L3)	5	Vertebrae (T12)
**7**	56	0	54	Vertebrae (T9, L1, and L2)
**8**	65	1 (L3)	49	Vertebrae (L1, and L2)
**9**	63	0	49	Shoulder (clavicle fracture)
**10**	57	1 (T8)	2	Vertebrae (T7)
**11**	89	1 (hip fracture)	49	Ribs fracture
**12**	69	0	48	Vertebrae (T12 and L3) and sacrum
**13**	84	1 (hip fracture)	21	Wrist (radius fracture)
**14**	58	0	35	Wrist (radius fracture)
**15**	61	1 (hip fracture)	44	Wrist (radius fracture)
**16**	61	1 (L2)	18	Elbow fracture
**17**	60	1 (T8, T10, T11, L3, and L5)	36	Ribs fracture
**18**	85	1 (ribs)	32	Hand (metacarpal fracture)
**19**	75	1 (T9 and T10)	13	Vertebrae, pelvis, and ribs fracture
**20**	75	1 (humeral fracture)	34	Vertebrae (T7)
**21**	53	1 (wrist)	13	Ribs fracture
**22**	64	1 (L1 and L2)	18	Foot (metatarsal fracture)
**23**	54	0	10	Shoulder (humeral fracture)
**24**	73	0	6	Shoulder (clavicle fracture)
**25**	67	1 (wrist)	23	Hand (metacarpal fracture)
**26**	64	0	6	Pelvis fracture
**27**	54	1 (pelvis)	22	Foot (Calcaneal fracture)
**28**	70	0	12	Foot (metatarsal fracture)
**29**	64	0	5	Shoulder (humeral fracture)

0 = no fracture at baseline (control group patient).

1 = fracture at baseline (case group patient).

### WFI-Based PDFFs and Incident Clinical Fractures

The lack of an association between WFI-based femoral head, neck, and diaphyseal PDFFs and the incidence of clinical fractures is shown in Table 3. Stepwise regression analysis did not alter these findings, as presented in Table 4. Nevertheless, the variable “recent osteoporotic fractures” was significantly associated with the incidence of clinical fractures. Furthermore, as shown in Table 3 and confirmed by the stepwise regression analysis presented in Table 4, WFI-based lumbar spine PDFF was not associated with the incidence of clinical fractures.

**Table 3.** Association between proton density fat fraction using water-fat imaging-based MRI and incident clinical fractures

	N	Total	ICF	CE	HR (95% CI)	*P* value
Lumbar spine	195	195	29	7	0.95 (0.67 to 1.35)	0.77
Femoral head	195	188	27	7	1.13 (0.70 to 1.80)	0.61
Femoral neck	195	188	27	7	1.07 (0.71 to 1.63)	0.74
Femoral diaphysis	195	188	27	7	1.00 (0.68 to 1.47)	0.99

HR indicates hazard ratios on subdistribution of clinical fracture and are calculated for an increase of 1 SD using a Fine and Gray regression model considering 7 deaths as competing events.

Abbreviations: CE, competing events (ie, death); HR, hazard ratio; ICF, incident clinical fractures; MRI, magnetic resonance imaging; PDFF, proton density fat fraction.

**Table 4.** Association between proton density fat fraction using water-fat imaging-based MRI and incident clinical fractures adjusted on confounding factors (selected using a forward selection)

	HR	95% CI	*P* value
MODEL 1			
PDFF lumbar spine	0.94*^a^*	(0.64 to 1.37)*^a^*	0.75
BMD lumbar spine	0.66*^a^*	(0.42 to 1.03)*^a^*	0.063
MODEL 2			
PDFF femoral head	1.20*^a^*	(0.70 to 2.06)*^a^*	0.51
Recent osteoporotic fracture	2.45	(1.10 to 5.41)	**0**.**027**
Age	0.73*^a^*	(0.48 to 1.10)*^a^*	0.13
MODEL 3			
PDFF femoral neck	1.13*^a^*	(0.73 to 1.76)*^a^*	0.58
Recent osteoporotic fracture	2.45	(1.09 to 5.47)	**0**.**029**
Age	0.73*^a^*	(0.47 to 1.11)*^a^*	0.14
MODEL 4			
PDFF femoral diaphysis	1.03*^a^*	(0.70 to 1.53)*^a^*	0.87
Recent osteoporotic fracture	2.43	(1.08 to 5.46)	**0**.**031**
Age	0.75*^a^*	(0.49 to 1.14)*^a^*	0.17

HR indicates hazard ratios on subdistribution of clinical fracture and are calculated using a Fine and Gray regression model considering 5 deaths as competing events and confounders factors (age, BMD [lumbar spine or total hip]), CCI, number of falls in the last 12 months, and recent osteoporotic fracture [less than 12 months old]) selected using a forward stepwise approach using a selection criterion of *P* < .20. Bold values are statistically significant values.

Abbreviations: BMD, bone mineral density; HR, hazard ratio; PDFF, proton density fat fraction.

^*a*^Calculated for an increase of 1 SD.

### MRS-Based PDFF, aLUL, and Incident Clinical Fractures

¹H-MRS was performed at the L3 vertebral level in 113 participants. The absence of a relationship between MRS-based L3 PDFF and L3 aLUL and the occurrence of clinical fractures is shown in Table 5. The stepwise regression analysis did not alter these findings, as shown in Table 6. ¹H-MRS was also performed at the femoral neck in a subset of 115 participants, yielding similar results.

**Table 5.** Association between proton density fat fraction and aLUL using ¹H-MRS and incident clinical fractures

	N	Total	ICF	CE	HR (95% CI)	*P* value
L3 PDFF	113	108	14	5	1.02 (0.58 to 1.77)	0.95
L3 aLUL	113	107	13	5	1.21 (0.61 to 2.42)	0.58
Femoral neck PDFF	115	113	14	5	0.84 (0.50 to 1.42)	0.52
Femoral neck aLUL	115	111	14	5	0.78 (0.51 to 1.20)	0.25

HR indicates hazard ratios on subdistribution of clinical fracture and are calculated for an increase of 1 SD using a Fine and Gray regression model considering 5 deaths as competing events.

Abbreviations: aLUL, apparent lipid unsaturation level; CE, competing events (ie, death); ^1^H-MRS, proton magnetic resonance spectroscopy; ICF, incident clinical fractures; PDFF, proton density fat fraction.

**Table 6.** Association between proton density fat fraction and aLUL using ¹H-MRS and incident clinical fractures adjusted on confounding factors (selected using a forward selection)

	HR	95% CI	*P* value
MODEL*^a^*			
L3 PDFF	1.10	(0.61 to 1.98)	0.75
BMD lumbar spine	0.63	(0.35 to 1.14)	0.13
MODEL 2			
L3 aLUL	0.83	(0.42 to 1.63)	0.58
BMD lumbar spine	0.62	(0.33 to 1.17)	0.14
MODEL 3			
Femoral neck PDFF	0.81	(0.50 to 1.32)	0.40
BMD femoral neck	0.74	(0.51 to 1.05)	0.091
MODEL 4			
Femoral neck aLUL	0.65	(0.36 to 1.14)	0.13
BMD femoral neck	0.68	(0.44 to 1.04)	0.07

HR indicates hazard ratios on subdistribution of clinical fracture and are calculated using a Fine and Gray regression model considering 5 deaths as competing events and confounders factors (age, BMD [lumbar spine or total hip], CCI, number of falls in the last 12 months, and recent osteoporotic fracture [less than 12 months old]) selected using a forward stepwise approach using a selection criterion of *P* < .20.

Abbreviations: aLUL, apparent lipid unsaturation level; CE, competing events (ie, death); ^1^H-MRS, proton magnetic resonance spectroscopy; ICF, incident clinical fractures; PDFF, proton density fat fraction.

^*a*^Calculated for an increase of 1 SD

## Discussion

### Main Findings

The research conducted in this ADIMOS ancillary study found no relationship between the WFI-based proximal femur, including the femoral head, neck, and diaphyseal regions, or lumbar spine PDFFs, and the incidence of clinical fractures. The same was confirmed for MRS-based PDFF, aLUL, and incident clinical fractures.

### Comparison With Existing Literature

There are few studies on the association between BMAT and fractures [12, 14-17]. In cross-sectional studies, (morphometric) VFs might be associated with a higher BMFF, independent of BMD (areal and volumetric) [12, 14, 20, 21]. To date, only 2 prospective studies, using the same cohort, evaluating the relationship between lipid content and composition at the lumbar spine, and fractures, have been published [15, 16]. In the AGES-Reykjavik study, no relationship was found between BMFF, incident radiographic VFs, and incident clinical fractures in either men or women during a mean follow-up of 3.3 years [15]. The results of our study are consistent with these findings. However, Woods et al found an association between higher levels of unsaturated lipids and a lower risk of incident radiographic VFs [16]. Fractures were identified through medical records for up to 8.8 years of follow-up, which is much longer than in our study. Wood et al also highlighted in the AGES-Reykjavik cohort a lower risk of incident clinical fractures in men, but not in women [16].

To the best of our knowledge, no previous investigations of BMAT and clinical incident fractures in elderly individuals have utilized ¹H-MRS and/or WFI to assess proximal femoral PDFF. Nevertheless, we did not uncover any association between the lipid content and composition measurements evaluated by MRI and incident clinical fractures, either at the lumbar spine or proximal femur.

### Clinical Significance

Diagnosing osteoporosis and predicting fracture risk depend on case-finding strategies that involve evaluating BMD using DXA and assessing the risk factors for osteoporosis. However, most fractures occur in individuals who have not been diagnosed with osteoporosis by BMD screening and have few risk factors [22]. Enhanced methods for identifying individuals with the greatest risk of fracture would enable the treatment of patients who would likely have the most favorable benefit-to-risk profiles, ultimately reducing the burden of fractures [23]. It has been postulated that noninvasive quantitative assessment of BMAT using MRI may improve the prediction of fractures. Although research on BMAT has gained momentum in recent years, it is essential to recognize that despite 4 decades of progress, MRI is still far from being widely adopted as a routine tool for evaluating BMAT as a biomarker of fracture risk in the elderly population [2]. Our study is not in favor of using BMAT imaging beyond BMD and risk factors of osteoporosis to assess the risk of fractures in postmenopausal women.

### Strengths and Limitations

The strengths of this study lie in the fact that (i) our study population is homogeneous, comprising exclusively postmenopausal women to prevent heterogeneity due to sex and menopausal status; (ii) BMAT was measured both at the lumbar spine and the proximal femur with 2 different MRI techniques that have been previously demonstrated to be roughly equivalent; and (iii) MRI and DEXA images were acquired on the same machines.

Our study has certain limitations. First, the cohort was limited to postmenopausal women aged ≥ 45 years; therefore, our results may not apply to younger women or men. The prevalence of osteoarthritis is notably high in this population, potentially leading to an overrepresentation of this comorbidity among postmenopausal women. Another limitation is the relatively small sample size, which reduced the statistical power to detect an association between PDFF and incident clinical fractures. A notable limitation is the reliance on self-reported fractures collected through telephone interviews, without systematic morphometric fracture evaluation during follow-up. To mitigate this limitation, 2 experienced rheumatologists conducted rigorous interviews and excluded any fracture events that lacked sufficient precision or clarity. While this approach likely reduced misclassification, it may have led to a slight underestimation of the fracture rate. However, a potential recall bias might have influenced the results. Furthermore, the exact date of incident fracture could not be identified for each patient. The use of AOM during the follow-up period likely contributed to a reduction in the number of observed incident clinical fractures. While clinically beneficial, it may have artificially obscured the relationship between PDFF measurements and fracture risk. Finally, the follow-up period was relatively short in our study, indicating the need for prospective follow-up studies over longer durations.

## Conclusions

In conclusion, the hypothesis that noninvasive measurements of lipid content and composition using MRI can improve the identification of postmenopausal women at risk of osteoporotic fractures has not been validated in this study. Further studies are necessary in larger cohorts and longer follow-up periods using the international BMAS methodological recommendations [11], and these studies should report measurements of proximal femur PDFF rather than vertebral PDFF alone.

## Data Availability

Original data generated and analyzed during this study are included in this published article.

## Abbreviations

aLUL: apparent lipid unsaturation level
BM: bone marrow
BMAS: International Bone Marrow Adiposity Society
BMAT: bone marrow adipose tissue
BMD: bone mineral density
BMFF: bone marrow fat fraction
CCI: Charlson Comorbidity Index
DXA: dual-energy x-ray absorptiometry
MRI: magnetic resonance imaging
MRS: magnetic resonance spectroscopy
PDFF: proton density fat fraction
VF: vertebral fracture
WFI: water-fat imaging
